# Molecular characterization and genotype shift of dengue virus strains between 2001 and 2014 in Guangzhou

**DOI:** 10.1017/S0950268816002429

**Published:** 2016-12-06

**Authors:** L.Y. JIANG, Q. L. JING, Y. LIU, Y. M. CAO, W. Z. SU, D. BIAO, Z. C. YANG

**Affiliations:** Guangzhou Center for Disease Control and Prevention, Guangdong, China

**Keywords:** Dengue virus, infectious disease, molecular epidemiology, phylogenetic analysis

## Abstract

We studied the evolution, genotypes, and the molecular clock of dengue virus serotype 1 (DENV-1), between 2001 and 2014 in Guangzhou, China. The analysis of the envelope (E) gene sequences of 67 DENV-1 strains isolated in Guangzhou, together with 58 representative sequences downloaded from NCBI, have shown shifts in viral genotypes. The genotype changed several times, from genotype I to IV in 2002, from IV to I in 2005, and from I to V in 2014. These genotype shifts may be the cause of DENV outbreaks. The diversity of genotypes and clades demonstrates a high risk of future outbreaks in Guangzhou. The mean rate of virus nucleotide substitution in Guangzhou was determined to be 7·77 × 10^−4^ per site per year, which represents a medium substitution rate compared to two other countries. Our research can point to different ancestors of the isolated strains, which may further reveal the different origins and transmission of DENV-1 strains in Guangzhou.

## INTRODUCTION

Dengue fever (DF) is a mosquito-borne viral disease, which is caused by dengue virus (DENV) belonging to the genus *Flavivirus,* family Flaviviridae. In the last few decades, DF has expanded throughout the world, and it is estimated that there are tens of millions of DF cases globally. Currently, the highest incidence of this disease is in Southeast Asia and the Western Pacific [1]. DENV is an enveloped virus, with an 11-kb, positive-sense single-stranded RNA genome. The genome encodes a single open reading frame and can be translated into three structural proteins, core (C), premembrane/membrane (prM/M), and envelope (E), and seven non-structural proteins, NS1, NS2a, NS2b, NS3, NS4a, NS4b, and NS5. DENVs are separated into four serotypes and several genotypes, according to the sequence of the E gene. The most recently identified serotype, serotype 5, was reported in October 2013, and was isolated from the serum sample of a 37-year-old farmer in Malaysia, collected in 2007. Although it remains unclear why serotype 5 emerged, it represents the first new serotype identified in the last half century [2, 3].

In China, DF was recorded for the first time in Xiamen, in 1873. No DENV strain had been isolated from a patient's sera before 1978, when a serotype 4 strain was isolated in an outbreak in Fuoshan, Guangdong. Since then, Guangdong, Guangxi, Fujian, Zhejiang, and Hainan provinces, along the southeast coast of China, have been affected by DF outbreaks [4–6]. Since 1978, DF cases have been reported almost every year in Guangdong. From 2001 to 2011, 3577 cases have been reported in Guangdong, and of those, 2536 cases (70·90%) were in Guangzhou. In 2014, in an historical outbreak in Guangdong, 45 189 cases were reported [7–9], with 37 340 cases (82·63%) in Guangzhou.

Guangzhou, the capital of Guangdong province, is located at 23° N, 113° E, close to the South China Sea. In 2013, according to the annual report of Guangzhou Municipal Bureau of Statistics [10], Guangzhou had a population of 12·93 million and a subtropical climate. Its humid climate, population demographics, and socio-economic conditions, such as the rapid growth of population, rural–urban migration, and overcrowded areas where *Aedes* mosquito larvae thrive, provide the ideal circumstances for DF transmission and spread. The first outbreak of dengue in Guangzhou was reported in 1978. From 2001 to 2014, three large outbreaks occurred in Guangzhou, in 2002, 2006, and 2014, with 1424, 765, and 37 340 cases, respectively [11].

Although four serotypes of DENV have been obtained during the Guangzhou epidemics from 2001 to 2014, serotype 1 has been shown to be the dominant serotype [12]. Unlike other serotypes, DENV-1 was detected almost every year and isolated most frequently in DF epidemics. Therefore, investigating the phylogenetic history of DENV-1 and the molecular clock dating, using the isolation time as the calibration point for the establishment of the date of virus introduction may be useful for understanding how different genotypes were established in Guangzhou and how they dispersed.

## METHODS

### DF patient definition

All DF cases were determined according to the unified diagnostic criteria, Diagnostic Criteria for Dengue Fever (WS216-2008) [13], issued by the Chinese Ministry of Health, and reported to the online Notifiable Infectious Disease Report System (NIDRS). According to these criteria, DF can be determined clinically or by laboratory analyses. The suspected DF cases were identified by local experienced physicians, according to the clinical manifestations and epidemiological exposure history, and further clinical diagnoses were provided. The patients that were clinically diagnosed with DF and presented with a fourfold increase in specific IgG antibody titre, positive PCR test, or a positive virus isolation and identification test, were considered laboratory-confirmed patients.

### Virus strains

DENV strains used in this study were isolated from the sera of the patients collected by the Guangzhou Center for Disease Control and Prevention (Guangzhou CDC) between 2001 and 2014 in Guangzhou, China. Serum samples were diluted 50-fold with RPMI-1640 (Gibco, USA) and used to inoculate a C6/36 cell monolayer, which was incubated at 28 °C for 7–10 days. DENV-positive cell cultures, showing typical cytopathic effects (CPE), were verified by the indirect immunofluorescence test. Negative cell cultures were inoculated into a new C6/36 cell monolayer. The cells inoculated with serum samples that showed no CPE after three generations were considered negative. The supernatants of the positive cultures were stored at −80 °C.

### RNA extraction, RT–PCR, and sequencing

RNA was extracted from cell culture supernatants using QIAamp Viral RNA Mini kit (Qiagen, Germany), according to the manufacturer's instructions. Next, RT–PCR was conducted using the SuperScript III One-Step RT–PCR System with Platinum *Taq* DNA polymerase (Invitrogen, USA). DEN750 (5′-CAAGAACCGAAACGTGGATG-3′) and DEN2639 (5′-TGTGGAAGCAAATATCACCTG-3′) primers were designed for the amplification of the full-length DENV-1 E gene. RT–PCR was performed using 1 μl of each primer at a concentration of 20 µm. The reaction was initiated at 50 °C for 30 min, followed by a denaturation step at 94 °C for 2 min, and 40 cycles of denaturation (94 °C for 30 s), primer annealing (52 °C for 30 s), and primer extension (72 °C for 2 min). The process ended with an extension step at 72 °C for 7 min. Amplified products were purified by agarose gel electrophoresis, and sequenced using an ABI 3730 Genetic Analyzer (Applied Biosystems, USA). Sequences were submitted to GenBank (http://www. ncbi.nlm.nih.gov).

### Phylogenetic analysis

Sixty-seven sequences from the isolated strains and 58 reference sequences from GenBank were aligned with ClustalW implemented in the MEGA software version 4.0 package [14]. Phylogenetic trees were constructed with the maximum parsimony and maximum-likelihood methods incorporated in the MEGA software version 5.0 package using the Kimura two-parameter model. The bootstrap value was 1000 replicates and only values >50 are presented.

### Molecular clock analysis

The rate of nucleotide substitution per site per year and time to the most recent common ancestor (tMRCA) were determined using Bayesian inferences implemented in BEAST v. 1.8.2 [15]. The data were analysed using the year of isolation as calibration points for the estimation of divergence time in years. A General Time-Reversible model combined with the Gamma + Invariant Site model was used as the nucleotide substitution model. A log-normal relaxed (uncorrelated) molecular clock was used for the analysis. A Markov Chain Monte Carlo (MCMC) chain was run for 10 000 000 steps. The parameter was sampled every 1000 steps and the first 1000 steps of each run were discarded. Tracer v. 1.6 (http://beast.bio.ed.ac.uk/Tracer) was used to analyse the log file. Tree Annotator v. 1.8.2 (http://beast.bio.ed.ac.uk/treeannotator) was used for choosing the maximum clade credibility tree, and FigTree v. 1.4.2 (http://beast.bio.ed.ac.uk/figtree) was used to view the annotated tree.

### Ethical standards

This study was approved by the Ethics Committee of the Guangzhou Center for Disease Control and Prevention, China. Written informed consent was obtained from all participants in the study. The authors assert that all procedures contributing to this work comply with the ethical standards of the relevant national and institutional committees on human experimentation and with the Helsinki Declaration of 1975, as revised in 2008.

## RESULTS

### Strains and sequences

A total of 123 strains of DENV-1 were isolated in the outbreaks occurring from 2001 to 2014, and their sequences were compared. One representative sequence from each year was used for analysis, if the sequences were identical. Sixty-seven sequences were used for phylogenetic analyses and molecular clock analysis (one strain in 2001, five strains in 2002, one strain in 2003, one strain in 2004, one strain in 2005, six strains in 2006, three strains in 2007, one strain in 2008, two strains in 2009, three strains in 2010, four strains in 2011, six strains in 2012, 11 strains in 2013, 22 strains in 2014). The sequences were submitted to the NCBI, and their respective accession numbers are shown in the phylogenetic tree (Supplementary Fig. S1). Fifty-eight reference sequences were downloaded from GenBank and analysed together with the isolated sequences.

### Phylogenetic reconstruction of DENV-1

The phylogenetic tree of the 67 isolated strains and 58 reference sequences are presented in Supplementary Figure S1. The isolated DENV-1 strains were segregated into three genotypes: genotype I (38 strains), genotype IV (10 strains), and genotype V (19 strains). Genotype I can be further divided into four clades, while genotype IV can be divided into two clades.

### Molecular clock

The total dataset for the molecular clock analysis consisted of 125 E gene sequences, 67 isolated strains, and 58 sequences downloaded from NCBI.

The maximum clade credibility tree is shown in Supplementary Figure S2. Under the log-normal relaxed clock (uncorrelated), the mean nucleotide substitution rate was shown to be 7·77 × 10^−4^ substitutions per site per year (95% highest probability density (HPD); 6·61 × 10^−4^ to 9·00 × 10^−4^). The results obtained using BEAST v. 1.8.2 showed that the tMRCA is about 92 years (95% HPD 82–102 years).

## DISCUSSION

Here, we report the prevalence of DENV in Guangzhou. Some studies suggest that DENV was imported from neighbouring countries to Guangzhou [16, 17], and ever since, all four DENV serotypes have circulated in all of these countries with equal frequency [18–22]. Four serotypes of DENV have been detected in Guangzhou, with at least three of them isolated from the imported patient cases [10]. However, DENV-1 serotype was shown to be the most frequent. It is known that the antibody against one virus serotype can protect against secondary infection with the same serotype. Therefore, after a certain period, it is difficult for the first predominantly circulating serotype to transmit efficiently in the population. However, the neutralizing antibody is unable to provide protection against the infections with heteroserotypes of DENV. This may explain the serotype shift, which occurs when the first predominantly circulating serotype is replaced by a different serotype [21]. However, from 2001 to 2014, for more than 10 years, DENV-1 has been the dominate serotype. Additional investigations are necessary in order to determine why serotype shift has not occurred, whether the Guangzhou population is more sensitive to DENV-1 compared to other serotypes, or whether this serotype has a shorter multiplication time.

Moreover, the antibody against a certain virus serotype may provoke the antibody-dependent enhancement, which can lead to dengue haemorrhagic fever (DHF) or dengue shock syndrome (DSS), following the subsequent heteroserotype infection. Therefore, after a lengthy period of serotype 1 epidemics, the epidemic of another serotype can lead to an increased incidence of DHF/DSS cases in Guangzhou.

As explained previously, antibodies are crucial for the serotype shifts and co-circulation. The E-coding region, a major target of neutralizing and enhancing antibodies, plays a significant role in antibody induction [21, 23]. The phylogenetic tree of the E gene showed that, before 2005, all DENV-1 strains were clustered in genotype IV, except for the strain isolated in 2001, which belongs to genotype I. After 2005, most of the strains were shown to be clustered in genotype I. However, since 2014, most of the isolated strains were shown to be genotype V. Every genotype shift led to an outbreak of DF. Genotype changes, from I to IV in 2002, from IV to I in 2005, and from I to V in 2014, were followed by large DF outbreaks in 2002, 2006, and 2014.

Similar to other RNA viruses, the degree of genetic diversity of DENV is high, not only between different serotypes, but also between the genotypes. This may be caused by mutations, recombination, or migration, and it can be intensified by the increase in the size and density of the human host population [24]. The nucleotide and amino acid differences found in the E region between the strains isolated in Guangzhou were shown to be 0–9·9% and 0–3·7%, respectively. It remains unclear if the 3·7% amino acid difference can lead to the genotype shift and co-circulation, but a study investigating neutralizing antibodies against DENV-1 demonstrated that only two of 79 human monoclonal antibodies stimulated by genotype II showed strong binding and neutralizing activity against all five DENV-1 genotypes [25]. This may explain the outbreaks following the genotype shifts in Guangzhou.

Phylogenetic tree analyses show that, before 2011, the strains isolated in the same year belonged to the same genotype, except for 2007. After 2011, the strains isolated in the same year belonged to different genotypes. In 2011, 2013, and 2014, genotypes I and V were detected, showing that different genotypes co-circulated at the same time. Higher genotype diversity leads to a higher rate of mutations, allowing the selection of more infective viruses. This may lead to the development of strains with an expanded range of pathogenic properties [26]. Even within the same genotype, the isolated strains were clustered in different clades, and the clade replacement was observed in many cases. Although the precise mechanisms underlying this diversity are unclear, DENV diversity may be shaped by complex interactions between the selective and stochastic forces. The selection pressure originating from different serotypes co-circulating in the population, density and biting activity of vector mosquitoes, and the stochastic genetic mutations, may play a role in the diversity of the clades.

BEAST v. 1.8.2 analyses show that the mean substitution rate in Guangzhou was 7·77 × 10^−4^ per site per year. Compared to the mean substitution rates of DENV-1 in New Delhi, India (6·28 × 10^−4^) [27], and Colombia (8·58 × 10^−4^) [28], the substitution rate was shown to be average in Guangzhou for the period 2001–2014. The DENV-1 MCMC tree showed that the strains isolated in Guangzhou have different ancestors, which may lead to the elucidation of the origin and transmission pathways of DENV-1 strains in Guangzhou.

Some studies suggest that clade diversity was associated with an increase in the abundance of serotype 1 [28–31], which may explain the historical dengue outbreak in Guangzhou in 2014, and this remains a high-risk area for DF outbreaks in the future.

## SUPPLEMENTARY MATERIAL

For supplementary material accompanying this paper visit https://doi.org/10.1017/S0950268816002429.
